# Endovascular treatment of pelvic venous congestion syndrome in a patient with duplication of the inferior vena cava and unusual pelvic venous anatomy: literature review

**DOI:** 10.1590/1677-5449.190017

**Published:** 2019-12-11

**Authors:** Marcelo Fernandes Lima, Ilídio Almeida Lima, Vanessa Heinrich-Oliveira

**Affiliations:** 1 Instituto de Flebologia Avançada, Manaus, AM, Brasil.; 2 Universidade Federal do Amazonas – UFAM, Manaus, AM, Brasil.; 3 Hospital das Clínicas, Universidade de São Paulo – USP, São Paulo, SP, Brasil.

**Keywords:** pelvic venous congestion, inferior vena cava duplication, pelvic varicose veins

## Abstract

Chronic pelvic pain affects approximately one-third of all women and is responsible for about 20% of all gynecological consultations. The authors report a rare case of symptomatic pelvic venous congestion in the presence of duplication of the inferior vena cava and inter-iliac communication through the right hypogastric vein that was treated via an endovascular approach with embolization of varicose pelvic veins. The published literature is reviewed.

## INTRODUCTION

Chronic pelvic pain (CPP) is defined as non-cyclical pain in the pelvic area lasting 3 months or more. It affects around 1/3 of all women and this symptom is responsible for up to 20% of all gynecological consultations. The most common etiologies of CPP include endometriosis, adenomyosis, pelvic inflammatory disease, and leiomyomas.^1^ Pelvic venous congestion (PVC), which can cause CPP, occurs when varicose veins develop around the many pelvic organs. Pelvic venous congestion is most often diagnosed in multiparous women, with clinical status typically characterized by non-cyclical lower abdominal or pelvic pains that are exacerbated by standing for long periods and by sexual intercourse, during the menstrual period and in pregnancy. Pain is characterized as heaviness, with associated symptoms, such as headaches, bloating, nausea, lower limb heaviness, lumbar pain, rectal discomfort, urinary urgency, lethargy, and depression. This set of symptoms associated with findings on physical examination of varicose veins involving vulva, perineum, and the posterior aspect of the tops of the lower limbs and buttocks is highly indicative of PVC and investigation should proceed to diagnostic confirmation with adequate imaging methods.^2^ The first report of duplication of the inferior vena cava (IVC) was published in 1916 in London (A case of double inferior vena cava. Lucas MF. J Anat 1916; 51:69-70).^3^ Since then, its incidence has been estimated in the range of 0.3 to 3% in several reports, with the great majority of cases being asymptomatic, with incidental diagnosis. Notwithstanding, knowledge of anatomic variants of the IVC is of vital importance, especially during retroperitoneal surgery and endovascular interventions.^3^

Although the studies available are of low quality, in terms of inappropriate study designs for assessing the efficacy of endovascular treatment with occlusion of varicose veins using coils and/or injection of sclerosant substances,^4^ the endovascular approach, with embolization of pelvic varicose veins and points of reflux, does appear to be the best method of treatment for PVC currently available.^5^ The minimally invasive character of endovascular procedures enables treatment of these patients in the office or day-hospital, reducing both the discomfort and the costs of a conventional surgical procedure. Reported therapeutic success rates of embolization to treat PVC range from 70 to 85%, with no negative impacts on the menstrual cycle, fertility, or ovarian hormone levels, with rates of complications estimated at 3.4 to 9%.^6^

## CASE REPORT

This bibliographic review was motivated by the case of a 27-year-old female patient who had never been pregnant and sought care complaining of burning pain, heaviness, and tiredness in lower limbs; symptoms that were exacerbated during her menstrual period. On physical examination of the patient, a large-caliber varicose vein was observed on the posterior-medial aspect of the upper third of the left thigh, in addition to varicules and telangiectasias distributed across both lower limbs. During history-taking, the patient described complaints compatible with PVC, such as dyspareunia and strong intensity pelvic pain during her menstrual period, in addition to recurrent urinary infections.

**Figure 1:**
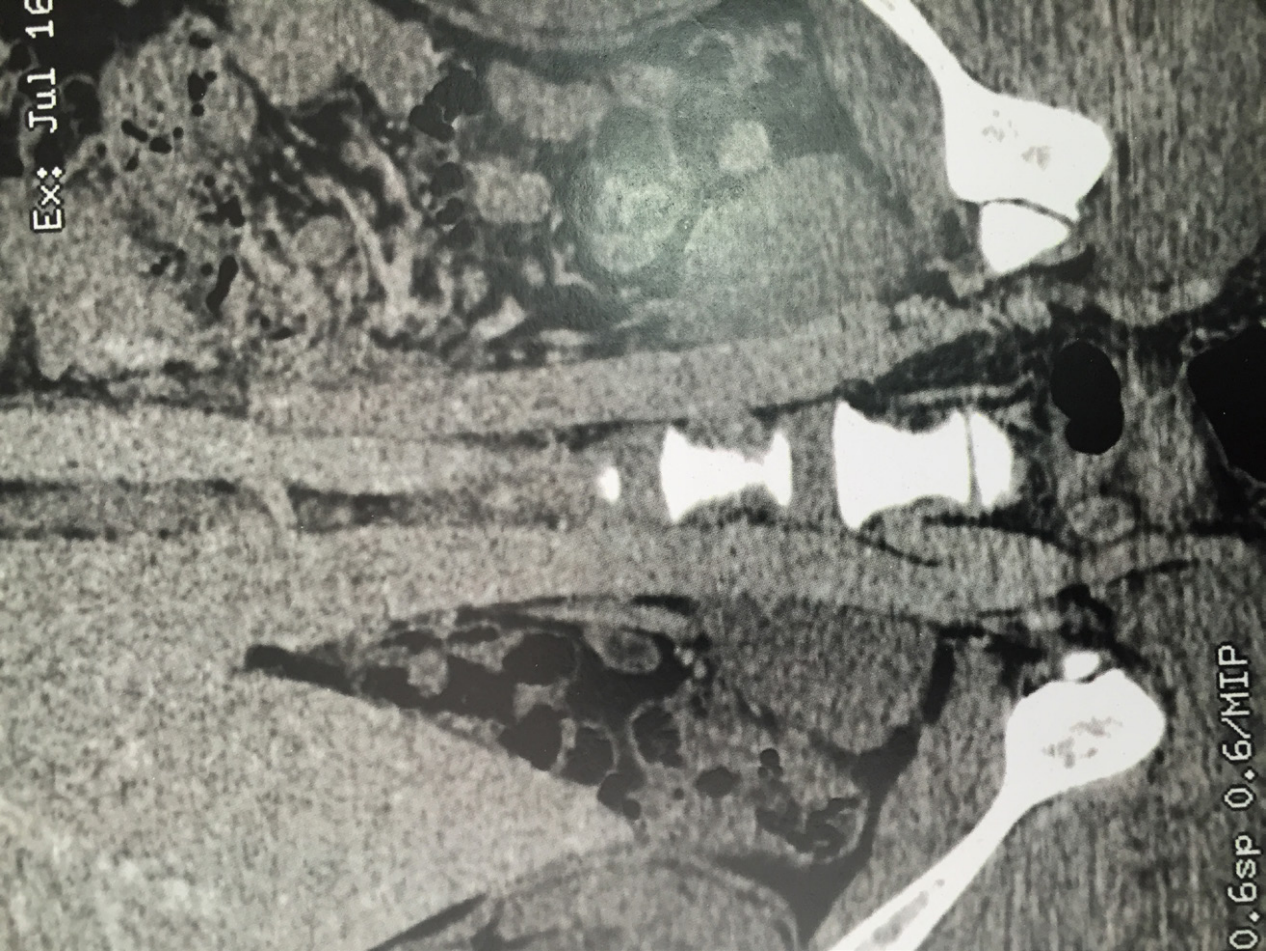
Angiotomography in venous phase, showing duplication of the inferior vena cava, forming a single vessel from the outflow of the left renal vein onwards.

**Figure 2:**
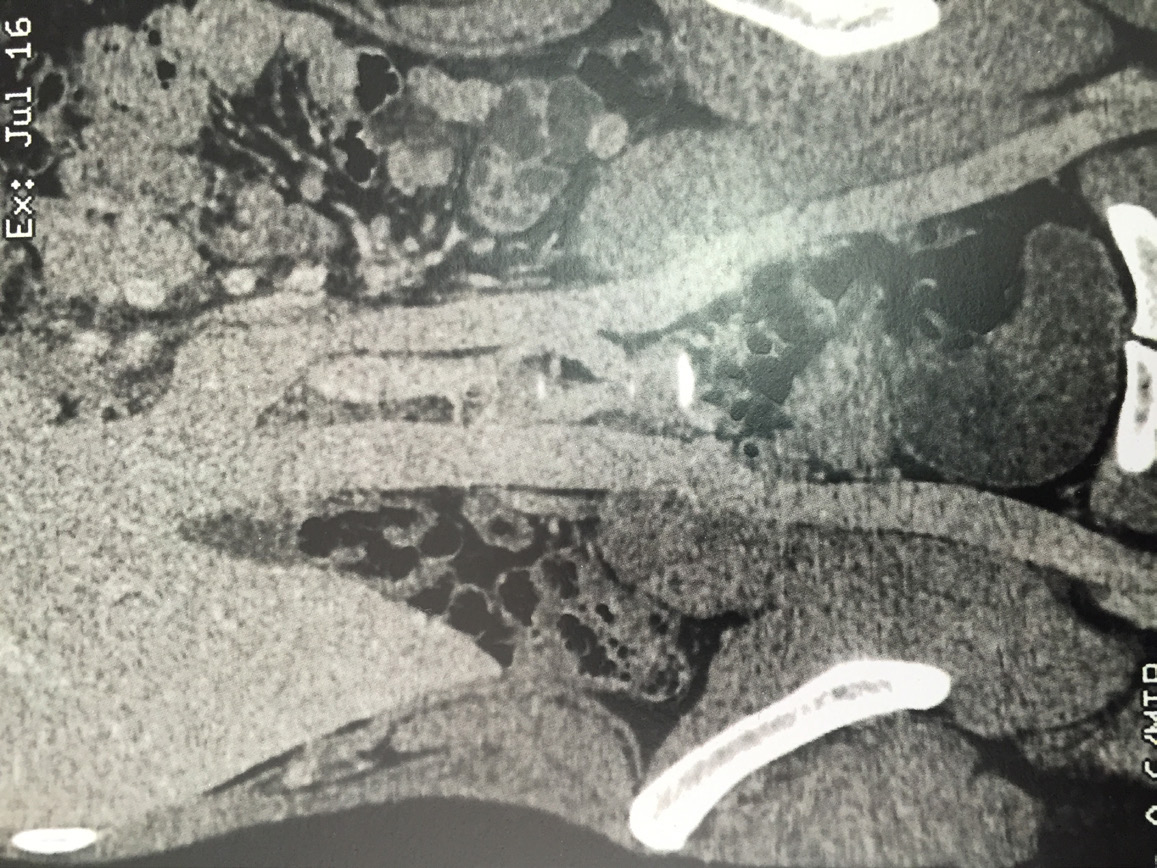
Angiotomography in venous phase, showing duplication of the inferior vena cava, forming a single vessel from the outflow of the left renal vein onwards.

Investigation with imaging exams was conducted with venous duplex scan of the lower limbs, which found no significant disorders of the superficial or deep venous systems, and with angiotomography in venous phase, which confirmed presence of pelvic varicose veins, and detected duplication of the infrarenal IVC, forming a single vessel from the outflow of the left renal vein onwards (Figures 1 and 2).

**Figure 3:**
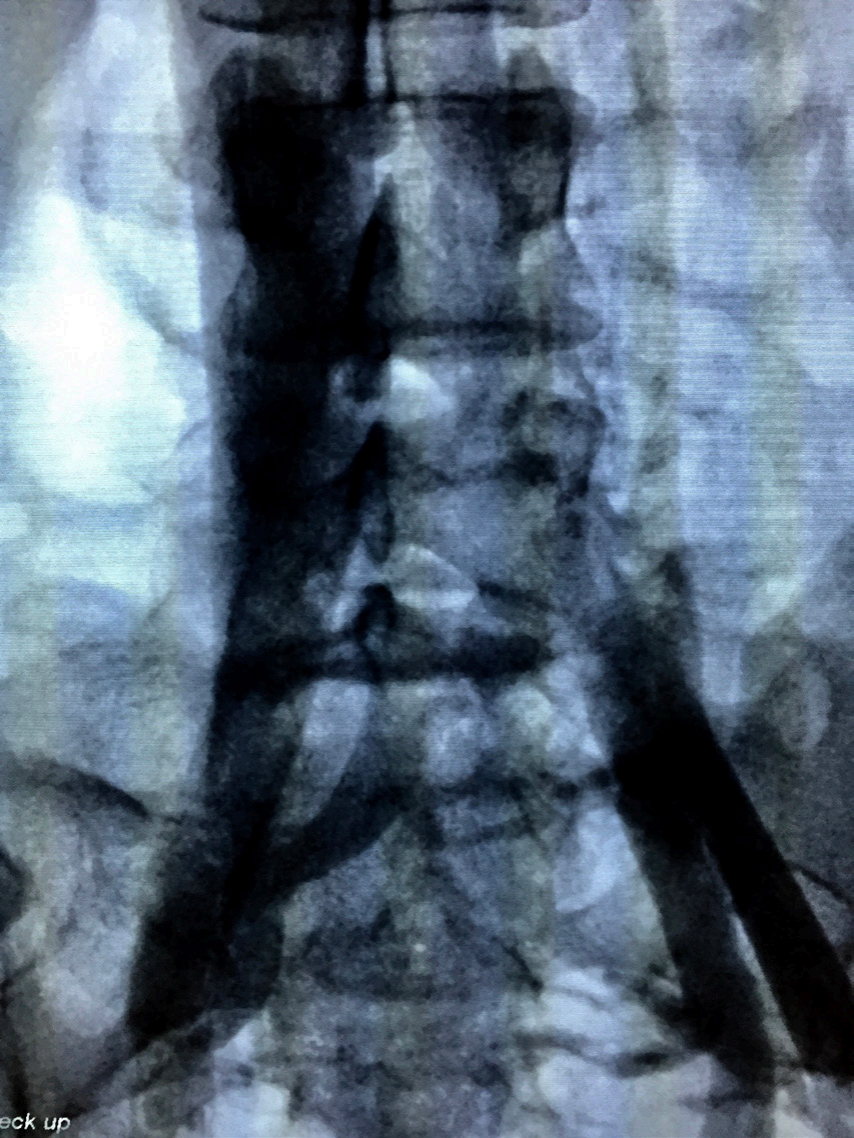
Angiography showing independent iliac systems and presence of an interiliac vein.

**Figure 4:**
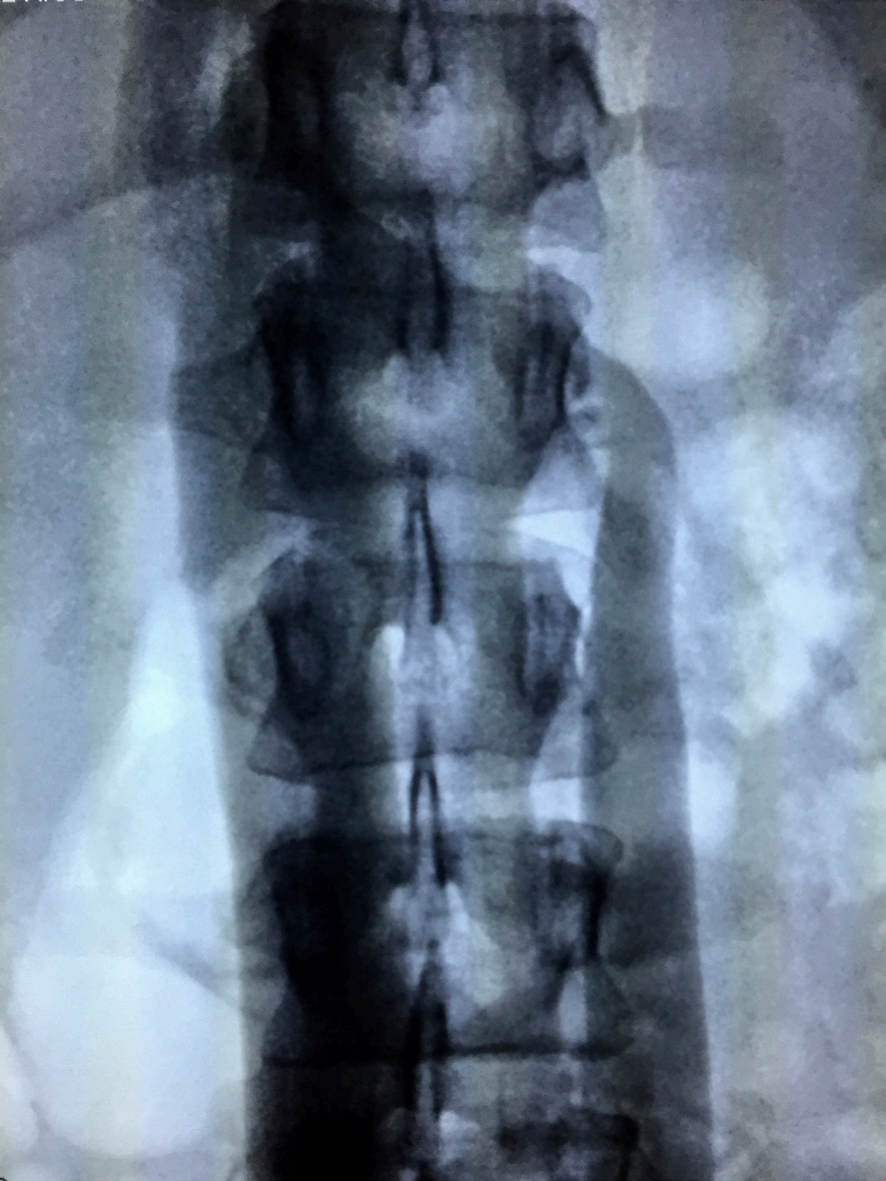
Formation of a single vessel with a junction between the two inferior vena cavas, after the outflow of the left renal vein.

**Figure 5:**
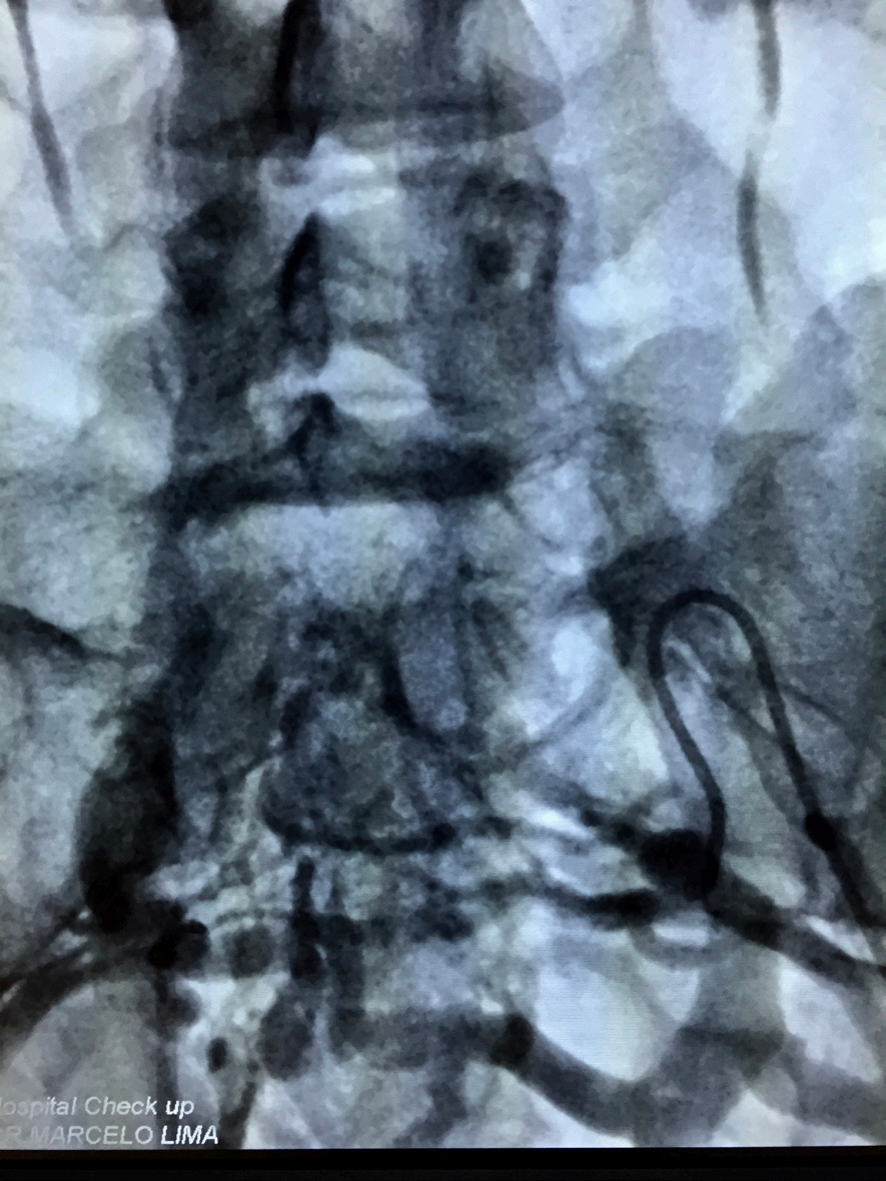
Presence of an interiliac vein communicating between the right and left iliac systems, with a considerable number of large caliber pelvic varicose veins.

**Figure 6:**
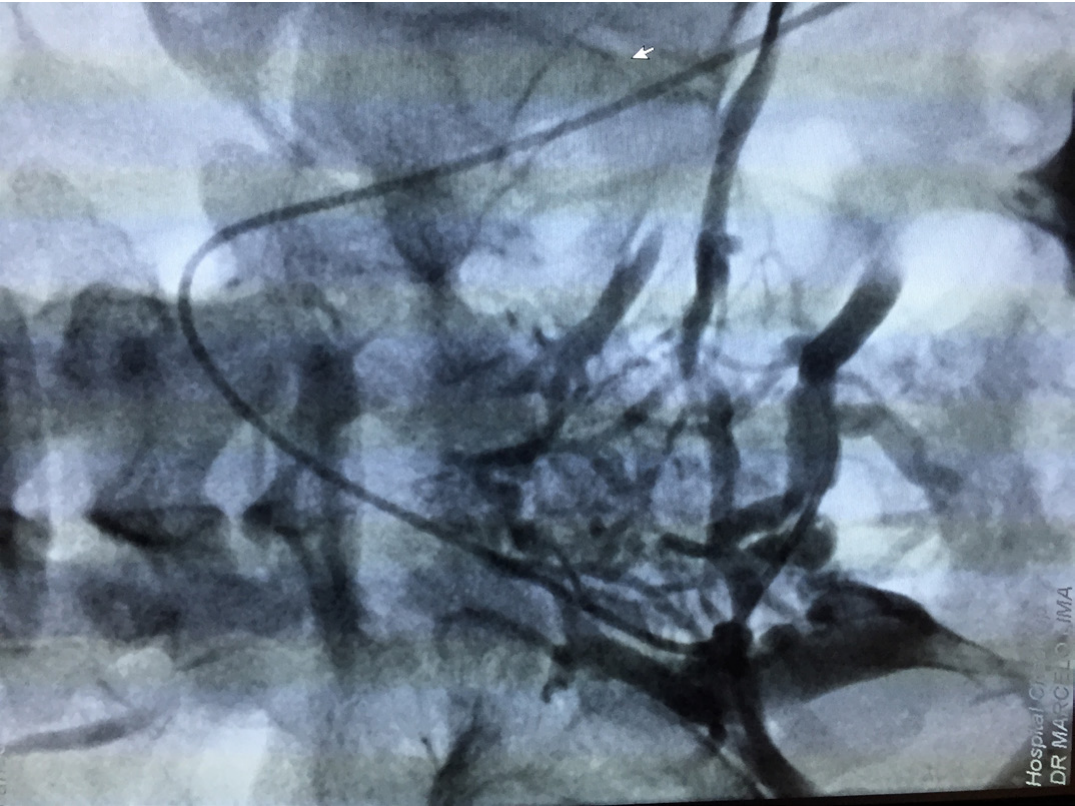
Selective catheterization of the interiliac vein and access to the right iliac system.

**Figure 7:**
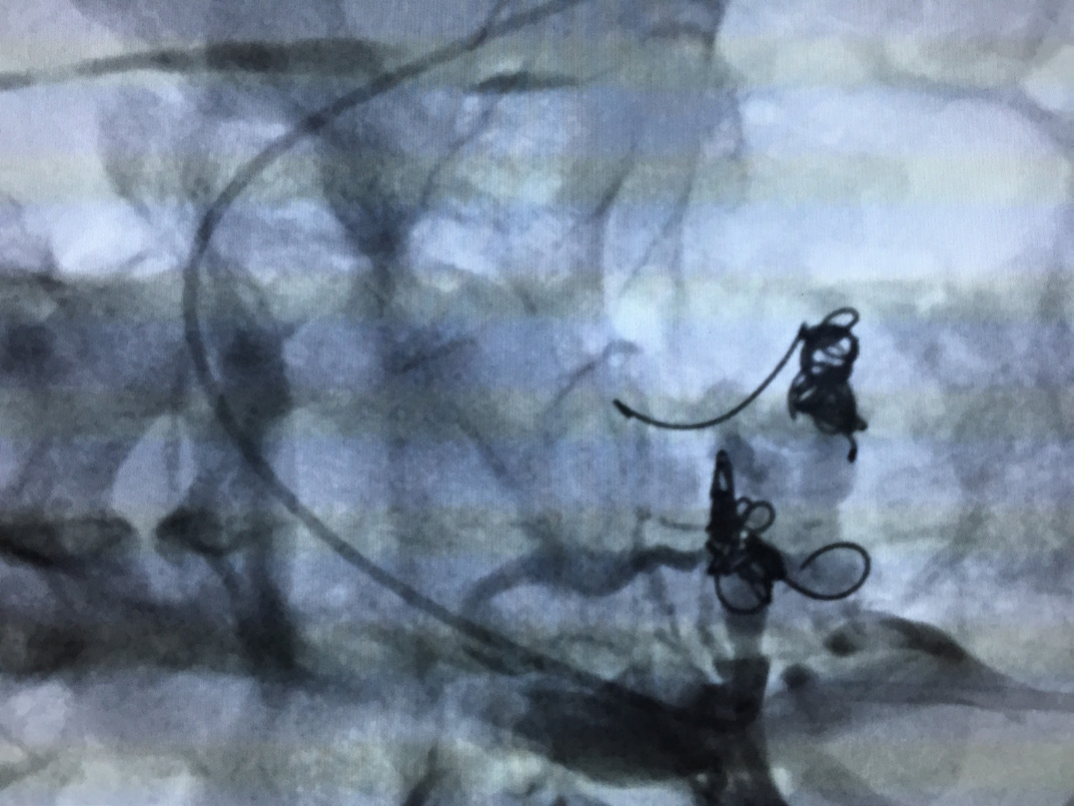
Deployment of fibered coils in tributary veins of the right iliac venous system after injection of polidocanol foam 1%.

**Figure 8:**
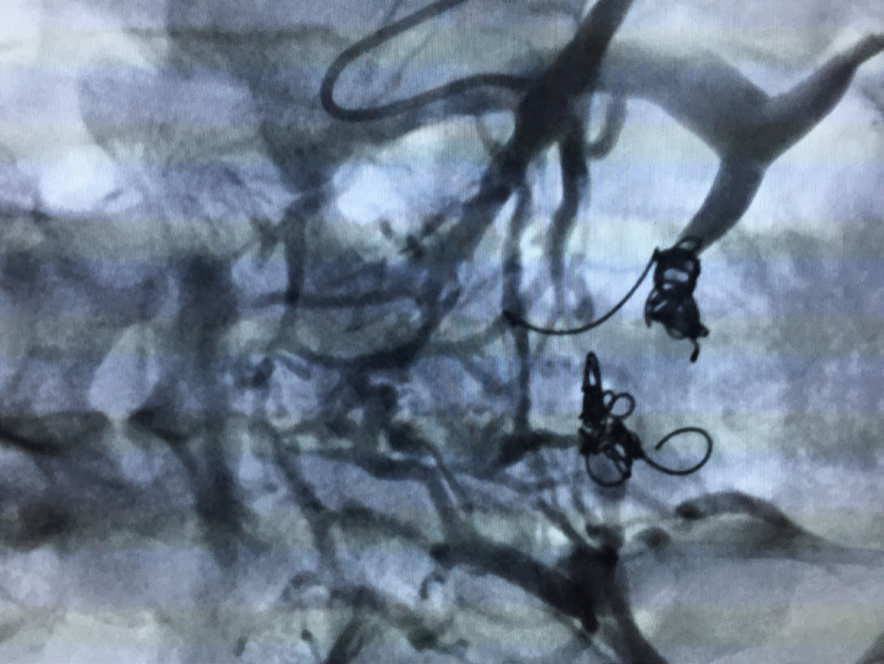
Selective catheterization of venous trunks feeding the varicose veins originating from the left internal iliac venous system.

**Figure 9:**
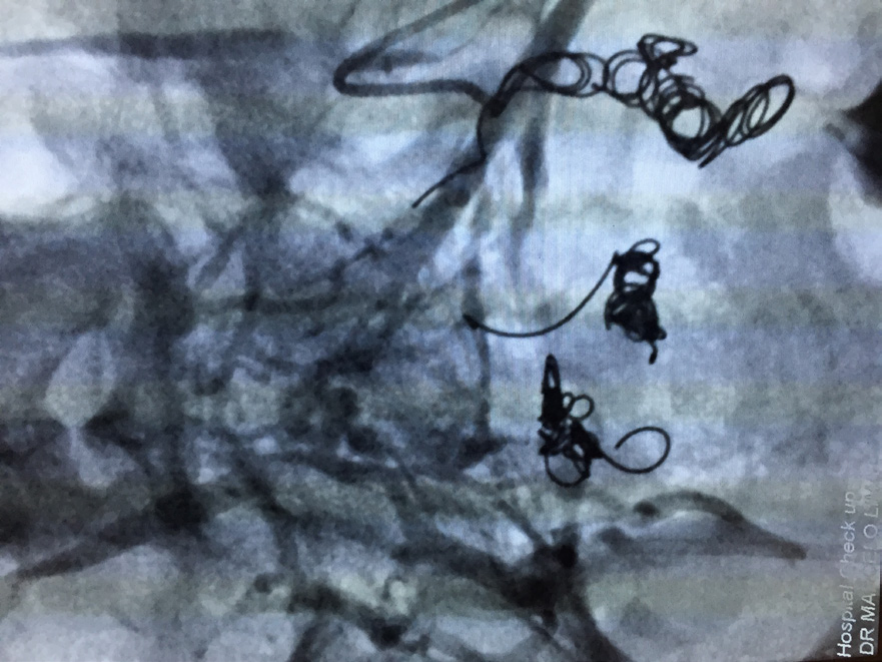
Deployment of fibered coils in tributary veins of the left iliac venous system after injection of polidocanol foam 1%.

**Figure 10:**
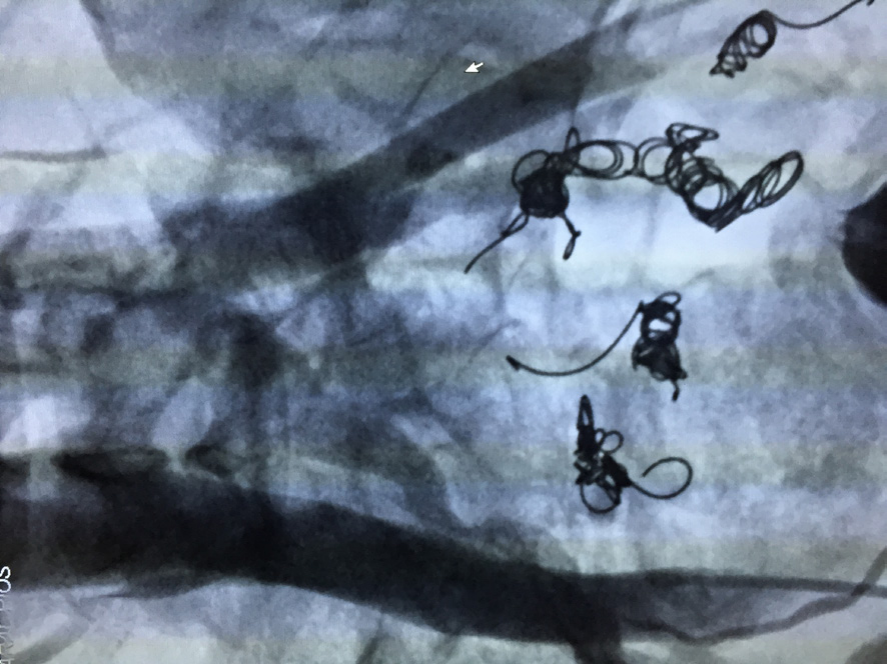
Postoperative control angiography showing occlusion of the pelvic varicose veins.

The treatment strategy planned was to eliminate proximal points of reflux by percutaneous embolization of pelvic varicose veins with deployment of fibered platinum coils, followed by chemical sclerotherapy of the varicose veins in the lower limbs. The first treatment step was accomplished via a percutaneous access to the left femoral vein, with pelvic and abdominal phlebography confirming duplication of the IVC, with each common iliac vein draining into the respective inferior vena cava (Figures 3 and 4) and an interiliac vein communicating between the right and left iliac systems, with a considerable number of large caliber pelvic varicose veins (Figure 5). The interiliac vein was selectively catheterized, accessing the right iliac system (Figure 6), polidocanol foam 1% was selectively injected into the varicose veins and fibered platinum coils were released into the venous trunks feeding the varicose veins originating from the right internal iliac venous system (Figure 7). Next, the left hypogastric vein was accessed, with superselective catheterization of the varicosed venous plexuses, further injection of polidocanol foam and release of fibered platinum coils into the venous trunks feeding the varicose veins originating from the left internal iliac venous system (Figures 8, 9, and 10). The immediate postoperative period was uneventful, with moderate pelvic pain, which responded promptly to administration of parenteral analgesia.

The patient reported that symptoms had ceased after embolization of the pelvic varicose venous plexuses and she remained asymptomatic and in follow-up 30 months after the intervention.

## DISCUSSION

The first time that incompetent dilated veins in the pelvis were linked to symptoms of PVC was in 1949, by Taylor. Since then, well-designed studies correlating PVC with CPP have not been published.^7^ Clinically, CPP is characterized by continuous or recurrent hypogastric or pelvic pain, with duration of at least 6 months and not limited to any specific period of the menstrual cycle or intercourse, or associated with pregnancy. An important factor is reduced quality of life and work incapacity, associated with significant mental, social, and physical burden, and etiology remains unconfirmed in 40 to 60% of cases, even after imaging studies and laparoscopy.^6^^,^ 7

Pelvic varicosities and CPP are typical findings of PVC, although women diagnosed with pelvic varicose veins may be asymptomatic, constituting a diagnostic challenge for gynecologists investigating CPP.^6^^,^ 8 Multiparity is a constant in patients with PVC, with complaints of dysmenorrhea and exacerbation of symptoms during or after coitus and when standing for long periods. The origin of PVC is very probably multifactorial and two factors appear to play an important role in genesis of cases. The first is valve incompetence caused by congenital absence of valves or presence of dysfunctional valves and the second is the around 60 times increase in pelvic venous capacity during pregnancy, due to mechanical compression by the gravid uterus and the vasodilatory action of progesterone, which can cause incompetence of venous valves, with subsequent venous hypertension and retrograde flow.^2^^,^ 9

It is estimated that 50% of patients with varicose veins have some type of predisposing genetic component. The FOXC2 gene was the first to be linked with the etiology of varicose veins, playing a key role in development and function of venous valves. Other studies have discovered associations between development of varicose veins and mutations of TIE2, NOTCH3, thrombomodulin, and transforming growth factor beta receptor, suggesting a genetic component in venous disease associated with PVC. Another factor that can also increase venous pressure and cause reflux with increased pelvic venous return via collaterals is mechanical compression of drainage veins, including nutcracker syndrome, May-Thurner Syndrome, endometriosis, fibromas, postoperative adherences, uterine leiomyomas, ovarian tumors, molar pregnancy, and mesenteric tumors. Anatomic tumors of the pelvic venous network can also contribute to development of PVC.^2^

Congenital anatomic variants of IVC have been reported with growing frequency in asymptomatic patients because of imaging study developments. The most common variant is duplication of the IVC, followed by left-sided IVC, and azygos continuation of the IVC. However, the pelvic anatomic variants of these anomalies and their relationships with the iliac veins and interiliac communicating veins have not received due attention, even though this knowledge is vital for reducing surgical risk and for defining access strategies in interventional radiology procedures.^10^

Embryogenesis of the IVC is a complex process involving development, regression, anastomosis, and substitution of the three major embryonic veins. The commonly accepted theory formulated to explain embryogenesis of the IVC is the foundation of embryological terminology. According to this theory, there are three pairs of embryonic veins and a variety of anastomoses between them that regress to form the IVC. They are named the posterior cardinal, the subcardinal, and the supracardinal veins.^11^

Embryogenesis of the IVC takes place between the 4th and 8th weeks of gestation. At this point, there are three groups of paired veins: the supracardinal, the posterior cardinal, and the subcardinal. These veins fuse and regress in succession until the IVC has been formed.^12^ The posterior cardinal vein is the first to emerge, in the posterior portion of the embryo. These veins regress, except for the distal portion, forming the iliac bifurcation. Next, the subcardinal veins emerge, anterior and medial to the posterior cardinal veins. The right subcardinal vein remains to form the suprarenal IVC, whereas the left regresses entirely. Subsequently, the supracardinal veins appear, dorsal to the subcardinal. The left supracardinal regresses and the right forms the infrarenal IVC. The normal IVC is therefore converted into a single, unilateral conduit on the right side, comprising four components: 1) the infrarenal segment, from the right supracardinal vein; 2) the renal segment, from anastomosis with the right supracardinal vein; 3) the suprarenal segment, from the right subcardinal; and 4) the hepatic segment, from the right hepatic vein. Several anastomoses develop between the different pairs of cardinal veins, with the right side dominating progressively. Anastomoses form between the two supracardinal veins, remnants of the distal extremities of the posterior cardinal veins, forming the left common iliac vein from the caudal segment. The right supracardinal and subcardinal systems produce the renal segment of the IVC and the renal veins.^13^ The duplicated IVC is therefore considered to be the result of persistence of both supracardinal veins.^3^

Knowledge of the countless variants of the pelvic venous anatomy is just as important as prior assessment of anatomic variants of the IVC for reducing iatrogenic potential and for deciding on access strategies for interventional radiology procedures.^10^

Formation of the iliac veins, including communicating vessels, during the initial stage of embryogenesis is complex and is still not entirely understood, although it has been reported that the internal iliac vein and its numerous visceral and parietal tributaries serve as important collateral routes in cases of obstruction of iliac-cava segments. Considering the variety of the descriptions in the literature, development of the iliac veins system may not be consistent, particularly with relation to the primordial origin of the hypogastric veins.^14^

**Figure 11:**
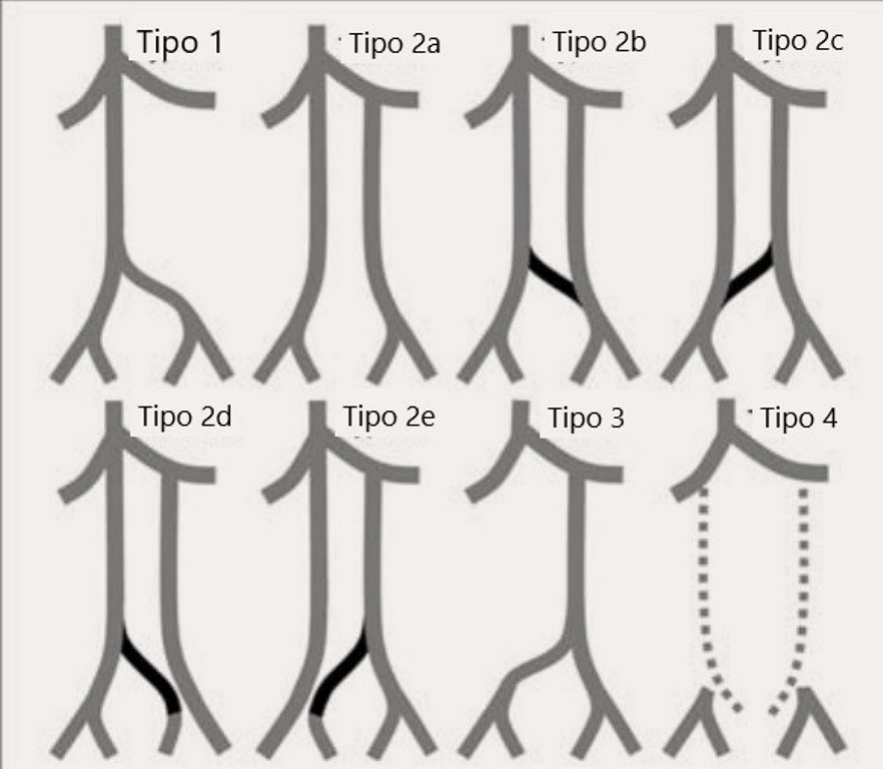
The Morita et al.^10^ classification of pelvic variants of inferior vena cava anomalies.

Morita et al.^10^ reviewed 11,719 abdominal and pelvic tomographic studies of 6,294 patients conducted from January 2004 to October 2006 in order to standardize classification of the different anatomic variants of the IVC and iliac veins. They observed 28 cases of IVC duplication, 6 cases of left-side IVC, 1 azygos continuation, and one absent infrarenal IVC. Using these tomographic findings, the authors proposed the following classification of pelvic variants of IVC anomalies, defining an interiliac communicating vein as a vein that drains blood from the iliac veins, including the common and external iliac and the hypogastric vein, to the side contralateral to the duplicated IVC^10^ (Figure 11):

Type 1: normal iliac confluence (including the azygos continuation);
Type 2a: IVC duplication, without interiliac communication;
Type 2b: IVC duplication, with interiliac communication from the left common iliac vein;
Type 2c: IVC duplication, with interiliac communication from the right common iliac vein;
Type 2d: IVC duplication, with interiliac communication from the left internal iliac vein;
Type 2e: IVC duplication, with interiliac communication from the right internal iliac vein;
Type 3: left-side IVC with normal or symmetrical iliac confluence;
Type 4: without iliac conjunction, with absent infrarenal IVC;

Posteriorly, Hayashi et al.^13^ proposed a classification based on the patterns of flow through the iliac veins, according to the paths of the hypogastric veins, dividing these patterns into three types^13^:

Type L: hypogastric vein draining to the ipsilateral external iliac vein;
Type M: interiliac communicating vein;
Type S: confluence of ipsilateral external iliac vein and the IVC.

In common with other vascular anomalies in the retroperitoneal space, a duplicated vena cava can be a cause both of incorrect diagnoses and of surgical complications. Surgeons, radiologists, oncologists, and urologists involved in therapeutic management of retroperitoneal space pathologies must not only have deep knowledge of the normal anatomy of this region, but also of their potential anatomic variants.^11^ Tong et al.^15^ reported two cases of vena cava filter implantation that were ineffective at preventing pulmonary embolization because of unknown IVC duplication in the patients.^15^ In Brazil, Malgor et al.^16^ report placing an IVC filter in a suprarenal position after cavography showed that the vessel was duplicated and also reported that the filter effectively prevented pulmonary embolism.^16^

Although several different diagnostic methods are under analysis for their potential to identify and diagnose PVC, including transvaginal ultrasound, computed tomography, and magnetic resonance,^4^^,^ 6 angiography is still the gold standard, for diagnosis both of PVC and of anatomic anomalies of the IVC and iliac veins, because it enables assessment of the different flow patterns and also allows treatment by coil embolization or decompression of venous trunks by stenting during the same intervention.^4^^,^ 8 ^,^ 17 Certain angiographic criteria should be present to confirm a diagnosis of PVC, specifically: reflux demonstrated by proximal injection of contrast into the ovarian vein with filling of the distal ovarian venous plexus, incompetent pelvic veins with 5 to 10 mm diameters, flow stasis in the ovarian venous plexus, with visualization of pelvic veins at the median line, vulvovaginal, and proximal thighs.^6^^,^ 17

There is no standardized treatment for PVC. All of the different treatment methods must be tailored to each patient on the basis of their symptoms and needs.^6^ Treatments that have been suggested for PVC include total abdominal hysterectomy, ligature or occlusion of pelvic varicose veins, and hormone therapy. Medroxyprogesterone acetate has been shown to temporarily reduce pain scores, but was associated with side effects such as weight gain and acne. Pelvic venous ligature is rarely used nowadays and total abdominal hysterectomy is unacceptable in young women.^7^

In contrast with conventional surgical procedures, endovascular venous procedures are minimally invasive and can eliminate points of reflux and varicose veins. They are generally conducted with local anesthesia and/or venous sedation and can be performed in the office or in a day hospital setting, reducing patients’ discomfort, anxiety, and costs.^6^ Currently, the method of choice for treatment of PVC appears to be percutaneous embolization. Although it has been suggested that foam sclerotherapy is sufficient to treat PVC, there are few data and little evidence to corroborate this. Placing fibered coils at the points of venous reflux appears to be the gold standard for endovascular treatment of PVC.^5^

With the objective of comparing the results of different occlusion devices, Guirola et al. conducted a randomized study assessing the results of plugs and fibered coils for occlusion of varicose veins and points of reflux. The study found that the mean cost of each coil was around 162 Euros, compared to 880 Euros per plug. Both groups were followed-up at 1, 3, 6, and 12 months, with therapeutic success assessed in terms of reduction or elimination of symptoms. In both groups, there was 96% technical success for cases of embolization of target veins, while in two patients it was not possible to embolize the right ovarian vein because of anatomic variants. Although the number of devices used was much lower in the group using plugs, the costs were nevertheless higher in this group. The group using coils had a larger number of major complications, with three migrations of coils to pulmonary arteries, compared to just one plug that migrated to a right pulmonary artery. The authors concluded that both plugs and coils are effective for occlusion of points of reflux and varicose veins, with significant improvement of the pelvic symptoms of PVC, with use of fewer plugs for total occlusion of the vessels, with the same clinical results obtained with fibered coils, and with reduced procedure time, fluoroscopy, and radiation dosages.^18^ In Brazil, Siqueira et al. conducted a retrospective study of 22 patients treated with embolization of periuterine varicose veins with fibered coils, observing clinical improvement in 76.9% of the patients, and this improvement was even more evident in patients with grade III reflux of the left ovarian vein. Minor complications, such as incapacitating pain, venous rupture without clinical repercussions, and postural hypotension, were observed in 18.2% of cases, and the authors concluded that percutaneous embolization is an effective and safe method for treatment of patients with PVC.^19^

Although data appear to confirm embolization as the treatment of choice, the quality of evidence is low. Daniels et al. conducted a systematic review of the literature using standard meta-analysis methods, attempting to estimate the overall proportion of patients with improved symptomology after embolization, using the proportions reported in the individual studies. They analyzed 21 studies of case reports and a low quality randomized study of 1,308 women. Although the objectives and techniques of embolization were clearly described, 1/3 of the studies did not specify the criteria for follow-up assessment nor how these data were collected. Inclusion of prospective studies only did not avoid 40% of the publications not making it clear what criteria were used to refer women for venography or define losses to follow-up. Significant initial improvements in pain were reported in around 75% of the patients subjected to embolization with gradual and sustained improvements over time and this result was observed in all of the studies that measured pain using visual analog scales. However, few data were included on the impact of treatment on menstruation, ovarian reserve, or fertility. The authors concluded that a well-designed randomized study is needed to definitively confirm efficacy of embolization for treatment of PVC.^19^

## CONCLUSIONS

Pelvic venous congestion is a common cause of CPP and one of the most underdiagnosed. Valuing symptoms during history-taking and a careful physical examination can lead to clinical suspicion, which should be followed by appropriate investigation including imaging exams for good treatment planning. Although currently available studies offer weak evidence because of poor design, the data and results reported appear to confirm the endovascular approach as treatment of choice in this scenario, since it enables potential points of venous reflux to be eliminated and varicose veins obliterated in a single intervention, resolving or considerably reducing patients’ complaints. It is also of great importance to conduct a careful examination of the pelvic venous anatomy, since this is a potential factor in morbidity and mortality during surgical interventions in this anatomic region.
