# Supplementary material for: Endovascular treatment of pelvic venous congestion syndrome in a patient with duplication of the inferior vena cava and unusual pelvic venous anatomy: literature review
Source: J Vasc Bras. 2019 Dec 11;19:e20190017. doi: 10.1590/1677-5449.190017 (PMC6944397; doi:10.1590/1677-5449.190017)
Supplement: Translation

# Translation: Tratamento endovascular de síndrome congestiva venosa pélvica em paciente com duplicação de veia cava inferior e anatomia venosa pélvica incomum: revisão bibliográfica

**Authors:** Marcelo Fernandes Lima, Ilídio Almeida Lima, Vanessa Heinrich-Oliveira

**Affiliations:** 1 Instituto de Flebologia Avançada, Manaus, AM, Brasil.; 2 Universidade Federal do Amazonas – UFAM, Manaus, AM, Brasil.; 3 Hospital das Clínicas, Universidade de São Paulo – USP, São Paulo, SP, Brasil.

**Keywords:** congestão venosa pélvica, veia cava inferior duplicada, varizes pélvicas

## Abstract

A dor pélvica crônica afeta aproximadamente 1/3 de todas as mulheres e é responsável por cerca de 20% de todas as consultas ginecológicas. Os autores relatam um raro caso de congestão venosa pélvica sintomática na presença de duplicação de veia cava inferior e comunicação interilíaca através de veia hipogástrica direita tratado com abordagem endovascular, por embolização das veias varicosas pélvicas e revisão da literatura publicada.

## INTRODUÇÃO

A dor pélvica crônica (DPC) é definida como dor em região pélvica, não cíclica, por tempo superior a 3 meses. Ela acomete cerca de um 1/3 de todas as mulheres, sendo esse sintoma responsável por até 20% de todas as consultas ginecológicas. Entre as principais etiologias de DPC, estão endometriose, adenomiose, doença inflamatória pélvica e leiomiomas^1^. Quando veias varicosas se desenvolvem em torno dos vários órgãos pélvicos, desenvolve-se um quadro de congestão venosa pélvica (CVP) que pode levar a um cenário de DPC. A CVP é mais frequentemente diagnosticada em mulheres multíparas, com quadro clínico tipicamente caracterizado por dor abdominal baixa ou pélvica não cíclica, exacerbada por ortostatismo prolongado, relação sexual, no período menstrual e na gravidez. A dor é caracterizada como tipo peso, com sintomas associados, como cefaleia, inchaço, náuseas, peso em membro inferior, dor lombar, desconforto retal, urência urinária, letargia e depressão. O conjunto de sintomas associado ao achado no exame físico de varizes em vulvas, períneo, face posterior de raiz de membros inferiores e nádegas é forte indicador da presença de CVP, devendo ser procedida a investigação para confirmação diagnóstica com métodos de imagens adequados^2^. O primeiro relato de duplicação de veia cava inferior (VCI) foi feito em 1916, em Londres (A case of double inferior vena cava. Lucas MF. J Anat 1916; 51:69-70)^3^. Sua incidência vem sendo estimada, desde então, entre 0,3 e 3% em vários relatos, com a grande maioria dos casos sendo assintomática, com diagnóstico incidental. No entanto, o conhecimento das variações anatômicas da VCI é de vital importância, especialmente durante cirurgias retroperitoniais e intervenções endovasculares^3^.

Apesar da baixa qualidade, devido ao desenho inadequado, dos estudos disponíveis avaliando a eficácia do tratamento endovascular com oclusão das veias varicosas com molas e/ou injeção de substâncias esclerosantes^4^, a abordagem endovascular com embolização das veias varicosas pélvicas e pontos de refluxo parece ser o melhor método de tratamento na atualidade para a CVP^5^. O caráter minimamente invasivo dos procedimentos endovasculares permite o tratamento desses pacientes em um regime ambulatorial ou de hospital-dia, reduzindo tanto o desconforto quanto os custos de um procedimento cirúrgico convencional. O sucesso terapêutico das embolizações para tratamento da CVP relatado varia entre 70 e 85%, sem impactos negativos no ciclo menstrual, fertilidade ou níveis hormonais ovarianos, com taxa de complicações estimada entre 3,4 e 9%^6^.

## RELATO DE CASO

A presente revisão bibliográfica foi motivada após atendimento de uma paciente do sexo feminino, nuligesta, 27 anos de idade, que procurou assistência queixando-se de dor urente, peso e cansaço em membros inferiores, sintomas esses exacerbados com piora durante o período menstrual. Ao exame físico da paciente, observou-se a presença de veia varicosa calibrosa em face póstero-medial de terço superior de coxa esquerda, além de varículas e telangiectasias distribuídas por ambos os membros inferiores. À anamnese, revelou queixas compatíveis com CVP, como dispareunia e dor pélvica de forte intensidade durante o período menstrual, bem como infecções urinárias de repetição.

A investigação com exames de imagens foi feita com *duplex scan* venoso de membros inferiores, que não evidenciou alterações significativas nos sistemas venosos superficiais e profundos, e com uma angiotomografia de fase venosa, que confirmou a presença de veias varicosas pélvicas, além de evidenciar VCI infrarrenal duplicada, que formava conduto único a partir da desembocadura da veia renal esquerda (Figuras 1 e 2).

**Figura 1:** Angiotomografia de fase venosa mostrando duplicidade de veia cava inferior, formando conduto único a partir de desembocadura de veia renal esquerda.

**Figura 2:** Angiotomografia de fase venosa mostrando duplicidade de veia cava inferior, formando conduto único a partir de desembocadura de veia renal esquerda.

A estratégia terapêutica traçada foi a eliminação dos pontos de refluxo proximais por embolização percutânea das veias varicosas pélvicas com implante de molas de platina fibradas e posterior escleroterapia química das veias varicosas dos membros inferiores. A etapa inicial do tratamento foi realizada por acesso percutâneo de veia femoral esquerda, com flebografia pélvica e abdominal confirmando a duplicidade da VCI, com cada veia ilíaca comum drenando para a respectiva cava inferior (Figuras 3 e 4), bem como a presença de veia interilíaca comunicando o sistema ilíaco direito ao esquerdo, com grande quantidade de varizes pélvicas calibrosas (Figura 5). Procedeu-se, então, a cateterização seletiva da veia interilíaca com acesso ao sistema ilíaco direito (Figura 6), injeção seletiva de espuma de polidocanol a 1% em veias varicosas e implante de molas de platina fibradas nos troncos venosos que nutriam as veias varicosas oriundas do sistema venoso ilíaco interno direito (Figura 7). Depois, procedeu-se o acesso seletivo de veia hipogástrica esquerda, com cateterização superseletiva dos plexos venosos varicosos, nova injeção de espuma de polidocanol e implante de molas de platina fibradas também nos troncos venosos nutridores das varizes oriundas do sistema venoso ilíaco interno esquerdo (Figuras 8, 9 e 10). O pós-operatório imediato da paciente transcorreu sem maiores intercorrências, com queixa de dor moderada pélvica, que cedeu prontamente com administração de analgesia parenteral.

**Figura 3:** Angiografia mostrando a presença de sistemas ilíacos independentes, com presença de veia interilíaca.

**Figura 4:** Formação de conduto venoso único com junção das duas veias cavas inferiores após desembocadura de veia renal esquerda.

**Figura 5:** Presença de veia interilíaca comunicando o sistema ilíaco direito ao esquerdo, com grande quantidade de varizes pélvicas calibrosas.

**Figura 6:** Cateterização seletiva da veia interilíaca com acesso ao sistema ilíaco direito.

**Figura 7:** Implante de molas fibradas em veias tributárias do sistema venoso ilíaco direito após injeção de espuma de polidocanol a 1%.

**Figura 8:** Cateterização seletiva dos troncos venosos nutridores das varizes oriundas do sistema venoso ilíaco interno esquerdo.

**Figura 9:** Implante de molas fibradas em veias tributárias do sistema venoso ilíaco esquerdo após injeção de espuma de polidocanol a 1%.

**Figura 10:** Angiografia de controle após o término do procedimento evidenciando oclusão das veias varicosas pélvicas.

A paciente relatou cessação dos sintomas após a embolização dos plexos venosos varicosos pélvicos, permanecendo assintomática em seguimento após 30 meses da intervenção.

## DISCUSSÃO

A primeira associação entre veias incompetentes e dilatadas na pelve com sintomas de CVP foi feita em 1949, por Taylor. Desde então, não houve publicação de estudos adequados correlacionando a CVP com a DPC^7^. A caracterização clínica de DPC se dá com o quadro de dor contínua ou recorrente, localizada em hipogástrio ou pelve, com duração de pelo menos 6 meses, não limitada a qualquer período do ciclo menstrual ou intercurso, nem associada com gestação. É um importante fator de redução de qualidade de vida e incapacidade laborativa, associada com um significativo fardo mental, social e físico, permanecendo sua etiologia não esclarecida entre 40 e 60% dos casos, mesmo após estudos de imagem e laparoscopia^6^^,^ 7.

Varicosidades pélvicas e DPC são achados típicos da CVP, no entanto, mulheres diagnosticadas com varizes pélvicas podem ser assintomáticas, o que caracteriza um desafio diagnóstico para o ginecologista investigando DPC^6^^,^ 8. A multiparidade é uma constante em pacientes com CVP, com queixas de dismenorreia e exacerbação dos sintomas durante ou após o coito e com ortostatismo prolongado. A origem da CVP é, muito provavelmente, multifatorial, com dois fatores parecendo ter importante papel na gênese do quadro. Primeiro, a incompetência valvular em decorrência de ausência congênita de válvulas ou presença de válvulas defeituosas e, segundo, o aumento em torno de 60 vezes da capacidade venosa pélvica durante a gestação pela compressão mecânica pelo útero gravídico e pela ação vasodilatadora da progesterona, o que pode levar à incompetência de válvulas venosas, com subsequente hipertensão venosa e fluxo retrógrado^2^^,^ 9.

Estima-se que 50% dos pacientes com veias varicosas tenham algum componente genético predisponente. O gene FOXC2 foi o primeiro a ser implicado na etiologia das veias varicosas e tem um papel-chave no desenvolvimento e funcionamento das válvulas venosas. Outros estudos descobriram associações entre o desenvolvimento de veias varicosas e mutações no TIE2, NOTCH3, trombomodulina e receptor do transformador do fator β de crescimento, o que sugere um componente genético para a doença venosa associada com CVP. Outro fator que também pode aumentar a pressão venosa e causar refluxo com aumento do retorno venoso pélvico via colaterais é a compressão mecânica de veias de drenagem, incluindo a síndrome de quebra-nozes, síndrome de May-Thurner, endometriose, fibromas, aderências pós-operatórias, leiomiomas uterinos, tumores ovarianos, mola hidatiforme e tumores mesentéricos. As variações anatômicas da rede venosa pélvica também podem contribuir para o desenvolvimento de CVP^2^.

As variações anatômicas congênitas da VCI têm sido cada vez mais relatadas em pacientes assintomáticos em decorrência dos avanços em estudos de imagem. A variação mais comum é a duplicidade de VCI, seguida pela VCI esquerda e continuação da ázigos. No entanto, as variantes anatômicas pélvicas dessas anomalias e suas relações com as veias ilíacas e comunicantes interilíacas não têm merecido a devida atenção, sendo vital o conhecimento dessas para a redução do risco cirúrgico e para determinar estratégias de abordagem em procedimentos de radiologia intervencionista^10^.

A embriogênese da VCI é um processo complexo envolvendo o desenvolvimento, a regressão, a anastomose e a substituição das três principais veias embrionárias. A teoria mais comumente aceita, que tenta explicar a embriogênese da VCI, é a base da Terminologia Embriológica. De acordo com ela, existem três pares de veias embrionárias com várias anastomoses entre elas que regridem e formam a VCI. São elas: as veias cardinais posteriores, as subcardinais e as supracadinais^11^.

A embriogênese da VCI ocorre entre a 4ª e a 8ª semana de gestação. Nesse ponto, existem três conjuntos de veias pareadas: as supracardinais, as cardinais posteriores e as subcardinais. Essas veias se fundem e regridem sucessivamente, até que a VCI esteja formada^12^. A veia cardinal posterior surge primeiro, na parte posterior do embrião. Essas veias regridem, exceto na porção distal, levando à formação da bifurcação ilíaca. Então, aparecem as veias subcardinais, anterior e medialmente às veias cardinais posteriores. A veia subcardinal direita permanece para formar a VCI suprarrenal, enquanto que a esquerda regride completamente. Subsequentemente, as veias supracardinais aparecem dorsalmente às subcardinais. A supracardinal esquerda regride, e a direita forma a VCI infrarrenal. A VCI normal é convertida, então, num conduto único, unilateral, posicionado à direita, consistindo em quatro componentes: 1) segmento infrarrenal, pela veia supracardinal direita; 2) segmento renal, pela anastomose da supracardinal direita; 3) segmento suprarrenal, pela subcardinal direita; e 4) segmento hepático, pela hepática direita. Várias anastomoses se desenvolvem entre os vários pares de veias cardinais, com o lado direito sendo progressivamente dominante. As anastomoses se formam entre as duas veias supracardinais, remanescentes da extremidade distal das veias cardinais posteriores, que formam a veia ilíaca comum esquerda no seu segmento caudal. Os sistemas supracardinais e subcardinais da direita produzem o segmento renal da VCI e as veias renais^13^. Portanto, a VCI dupla é considerada ser formada pela persistência de ambas as veias supracardinais^3^.

Tão importante quanto avaliar previamente as variações anatômicas da VCI é o conhecimento das inúmeras variações da anatomia venosa pélvica para diminuição do potencial iatrogênico e para determinar estratégias de abordagem em procedimentos de radiologia intervencionista^10^.

A formação das veias ilíacas, incluindo as comunicantes, durante o estágio inicial embrionário é complexa e permanece incerta, sendo relatado que a veia ilíaca interna e suas numerosas tributárias viscerais e parietais servem como importante via colateral nos casos de obstrução segmentar ilíaco-caval. Considerando a variedade de descrições na literatura, o desenvolvimento do sistema das veias ilíacas pode não ser consistente, particularmente em relação à origem primordial das veias hipogástricas^14^.

No intuito de padronizar as diferentes classificações das variações anatômicas da VCI e ilíacas, Morita et al.^10^ revisaram 11.719 estudos tomográficos abdominais e pélvicos em 6.294 pacientes entre janeiro de 2004 e outubro de 2006, encontrando 28 casos de duplicidade VCI, 6 de VCI à esquerda, 1 continuidade de ázigos e uma ausência de VCI infrarrenal. Com base nos achados tomográficos, os autores propuseram a seguinte classificação das variações pélvicas nas anomalias da VCI, sendo definida como veia comunicante interilíaca uma veia que drena sangue das veias ilíacas, incluindo ilíacas comum, externa e hipogástricas, para o lado contralateral da VCI duplicada^10^ (Figura 11):

**Figura 11:** Classificação de Morita et al.^10^ das variações pélvicas nas anomalias da veia cava inferior.

Tipo 1: confluência ilíaca normal (incluindo a continuação da ázigos);
Tipo 2a: duplicidade da VCI, sem comunicação interilíaca;
Tipo 2b: duplicidade da VCI, com comunicação interilíaca a partir da veia ilíaca comum esquerda;
Tipo 2c: duplicidade da VCI, com comunicação interilíaca a partir da veia ilíaca comum direita;
Tipo 2d: duplicidade da VCI, com comunicação interilíaca a partir da veia ilíaca interna esquerda;
Tipo 2e: duplicidade da VCI, com comunicação interilíaca a partir da veia ilíaca interna direita;
Tipo 3: VCI esquerda com confluência normal ou simétrica das ilíacas;
Tipo 4: sem conjunção ilíaca, com ausência de VCI infrarrenal;

Posteriormente, Hayashi et al.^13^ propuseram uma classificação baseada nos padrões de fluxo das veias ilíacas de acordo com o trajeto das veias hipogástricas, dividindo em três tipos esses padrões:

Tipo L: veia hipogástrica drenando na veia ilíaca externa ipsilateral;
Tipo M: veia comunicante interilíaca;
Tipo S: confluência da veia ilíaca externa ipsilateral e VCI.

A veia cava duplicada, junto com outras anomalias vasculares no retroperitônio, pode ser causa tanto de erro diagnóstico quanto de complicações cirúrgicas. Cirurgiões, radiologistas, oncologistas e urologistas envolvidos no manejo terapêutico de patologias do retroperitônio devem não só ter conhecimento profundo da anatomia normal dessa região, bem como de suas potenciais variantes anatômicas^11^. Tong et al.^15^ relataram dois casos de implante de filtro de veia cava que não foram eficazes em prevenir a embolização pulmonar pelo desconhecimento da duplicidade de VCI nos pacientes^15^. No nosso meio, Malgor et al.^16^ relataram um implante de filtro de VCI em posição suprarrenal, após a identificação em cavografia da duplicidade do vaso, filtro esse que se mostrou eficaz na prevenção da embolia pulmonar^16^.

Embora vários métodos diagnósticos venham sendo avaliados para identificar e diagnosticar a CVP, como ultrassom transvaginal, tomografia computadorizada e ressonância magnética^4^^,^ 6, a angiografia permanece como padrão-ouro para o diagnóstico tanto da CVP quanto das anomalias anatômicas da VCI e ilíacas por permitir avaliar os diferentes padrões de fluxo e também o tratamento por embolização com molas ou descompressão de troncos venosos por implante de *stents* vasculares no mesmo ato^4^^,^ 8 ^,^ 17. Alguns critérios angiográficos devem estar presentes para o diagnóstico da CVP, a saber: refluxo demonstrado por injeção proximal de contraste na veia ovariana com enchimento do plexo venoso ovariano distal, veias pélvicas incompetentes com 5 a 10 mm de diâmetro, estase do fluxo no plexo venoso ovariano, com visualização de veias pélvicas na linha mediana, vulvovaginais e proximais de coxas^6^^,^ 17.

Não há tratamento padronizado para a CVP. As diversas modalidades terapêuticas devem ser individualizadas com base nos sintomas e nas necessidades de cada paciente^6^. Os tratamentos sugeridos para a CVP incluem histerectomia abdominal total, ligadura ou oclusão de varizes pélvicas e terapia hormonal. O acetato de medroxiprogesterona tem mostrado aliviar temporariamente os escores de dor, mas foi associado a efeitos colaterais como ganho de peso e acne. A ligadura venosa pélvica é raramente realizada atualmente, e a histerectomia total abdominal é inaceitável em mulheres jovens^7^.

Em contraste com os procedimentos cirúrgicos convencionais, os procedimentos endovasculares venosos são minimamente invasivos e conseguem eliminar os pontos de refluxo e as veias varicosas. Geralmente, eles são realizados com anestesia local e/ou sedação venosa, podendo ser em caráter ambulatorial ou em regime de hospital-dia, reduzindo o desconforto, a ansiedade do paciente e os custos^6^. Atualmente, o método de escolha para tratamento da CVP parece ser a embolização percutânea. Embora tenha-se sugerido que a escleroterapia com espuma seja suficiente para tratar a CVP, poucos dados e evidências corroboram isso. O implante de molas fibradas nos pontos de refluxo venoso parece ser o padrão-ouro no tratamento endovascular da CVP^5^.

Com o objetivo de comparar os resultados entre diferentes dispositivos oclusivos, Guirola et al.^18^ fizeram um estudo randomizado avaliando os resultados do uso de *plugs* e molas fibradas para a oclusão de veias varicosas e pontos de refluxo. Nesse estudo, o custo médio de cada mola era de cerca de 162 euros e, de cada *plug*, 880 euros. Ambos os grupos tiveram seguimento de 1, 3, 6 e 12 meses, com sucesso terapêutico avaliado como diminuição ou eliminação dos sintomas. Em ambos os grupos, houve sucesso técnico de 96% dos casos na embolização das veias-alvo, sendo que, em dois pacientes, não foi possível a embolização da veia ovariana direita por variações anatômicas. Embora o número de dispositivos utilizado tenha sido muito menor no grupo em que se utilizaram *plugs*, o custo também foi mais elevado nesse grupo. O grupo utilizando molas teve maior número de complicações maiores, com três migrações de molas para artérias pulmonares, enquanto apenas um *plug* migrou para uma artéria pulmonar direita. Concluíram os autores que, tanto o uso de *plugs* quanto o de molas são eficazes para a oclusão dos pontos de refluxo e das veias varicosas, com a melhora significativa dos sintomas pélvicos da CVP, com a utilização de menos *plugs* para a total oclusão dos vasos, com os mesmos resultados clínicos obtidos com as molas fibradas, com tempo de procedimento, de fluoroscopia e doses de radiação também reduzidos^18^. No nosso meio, Siqueira et al.^8^, em estudo retrospectivo de 22 pacientes submetidas à embolização de veias varicosas periuterinas com molas fibradas, observaram melhora clínica em 76,9% das pacientes, sendo essa melhora ainda mais evidente em pacientes com refluxo grau III da veia ovariana esquerda. Complicações menores, como dor incapacitante, ruptura venosa sem repercussões clínicas e hipotensão postural, foram observadas em 18,2% dos casos, concluindo os autores que a embolização percutânea é um método eficaz e seguro para o tratamento de pacientes com CVP^19^.

Embora os dados pareçam corroborar a embolização como tratamento de escolha, a qualidade de evidências é baixa. Daniels et al.^19^, em uma revisão sistemática da literatura utilizando métodos padrão de metanálise, tentaram estimar a proporção geral de pacientes com melhora da sintomatologia após embolização, utilizando as proporções reportadas em estudos individuais. Foram analisados 21 estudos de relatos de casos e um estudo randomizado de baixa qualidade envolvendo 1.308 mulheres. Embora os objetivos e as técnicas de embolização tenham sido claramente detalhados, 1/3 dos estudos não especificou os critérios de avaliação no seguimento e como esses dados seriam coletados. A inclusão apenas de estudos prospectivos não impediu que, em 40% das publicações, não ficassem claro os critérios pelos quais mulheres foram referendadas para venografia, nem que a perda de seguimento também fosse claramente especificada. Foi relatada melhora inicial importante da dor em cerca de 75% das pacientes submetidas à embolização com melhora gradual e sustentada com o passar do tempo, sendo esse resultado observado em todos os estudos que mensuraram a dor em escalas análogas visuais. No entanto, poucos dados sobre o impacto do tratamento na menstruação, reserva ovariana ou fertilidade foram incluídos. Concluem os autores que se faz necessário um estudo randomizado adequado para corroborar definitivamente a eficácia da embolização no tratamento da CVP^19^.

## CONCLUSÃO

A CVP é uma causa frequente de DPC e uma das mais subdiagnosticadas. A valorização dos sintomas durante a anamnese e um exame físico cuidadoso podem levar à suspeita clínica, devendo haver uma correta investigação complementar que inclui exames de imagem para um adequado planejamento terapêutico. Embora os estudos atuais disponíveis contenham fracas evidências devido a desenho inadequado, os dados apresentados e os resultados obtidos parecem corroborar a abordagem endovascular como o tratamento de escolha nesse cenário, permitindo, ao mesmo tempo, eliminar os potenciais pontos de refluxo venoso e a obliteração de veias varicosas, com cessação ou diminuição importante das queixas apresentadas pelas pacientes. Também é de suma importância uma avaliação cuidadosa da anatomia venosa pélvica, haja vista essa ser um fator potencial de morbimortalidade durante intervenções cirúrgicas nesse território anatômico.
